# Sex bias in copy number variation of olfactory receptor gene family depends on ethnicity

**DOI:** 10.3389/fgene.2013.00032

**Published:** 2013-03-14

**Authors:** Farideh Shadravan

**Keywords:** copy number variation, sex bias, olfactory receptor genes, pseudogenes, ethnicity, mental retardation, Prader–Willi/Angelman syndromes, the 1000 Genome Project

## Abstract

Gender plays a pivotal role in the human genetic identity and is also manifested in many genetic disorders particularly mental retardation. In this study its effect on copy number variation (CNV), known to cause genetic disorders was explored. As the olfactory receptor (OR) repertoire comprises the largest human gene family, it was selected for this study, which was carried out within and between three populations, derived from 150 individuals from the 1000 Genome Project. Analysis of 3872 CNVs detected among 791 OR loci, in which 307 loci showed CNV, revealed the following novel findings: Sex bias in CNV was significantly more prevalent in uncommon than common CNV variants of OR pseudogenes, in which the male genome showed more CNVs; and in one-copy number loss compared to complete deletion of OR pseudogenes; both findings implying a more recent evolutionary role for gender. Sex bias in copy number gain was also detected. Another novel finding was that the observed sex bias was largely dependent on ethnicity and was in general absent in East Asians. Using a CNV public database for sick children (International Standard Cytogenomic Array Consortium) the application of these findings for improving clinical molecular diagnostics is discussed by showing an example of sex bias in CNV among kids with autism. Additional clinical relevance is discussed, as the most polymorphic CNV-enriched OR cluster in the human genome, located on chr 15q11.2, is found near the Prader–Willi syndrome/Angelman syndrome bi-directionally imprinted region associated with two well-known mental retardation syndromes. As olfaction represents the primitive cognition in most mammals, arguably in competition with the development of a larger brain, the extensive retention of OR pseudogenes in females of this study, might point to a parent-of-origin indirect regulatory role for OR pseudogenes in the embryonic development of human brain. Thus any perturbation in the temporal regulation of olfactory system could lead to developmental delay disorders including mental retardation.

## INTRODUCTION

Many human genetic disorders, including those related to developmental delay and intellectual disabilities, are well-known to show sex bias (Neul and Zoghbi, 2004; Rinehart et al., 2011). Recombination rates are also known to be different in males and females (Broman et al., 1998; Fledel-Alon et al., 2011). Furthermore, it has been shown that many human imprinted chromosomal regions are involved in sex-specific recombination (Paldi et al., 1995; Sandovici et al., 2006), and parent-of-origin deletion of imprinted regions are known to cause genetic diseases (Soejima and Wagstaff, 2005). As non-allelic homologous recombination takes place between interspersed duplicated sequences, it is proposed that copy number variation (CNV), the gain and/or loss of genomic materials, occurs through non-allelic homologous recombination (Hastings et al., 2009) suggesting a direct connection between CNV and recombination. Thus these various observations hint to the likelihood of a link between gender and CNV in normal people, as well as, those with genetic disorders, which are both the subjects of this study.

However, the scope of this study was not set at the genomic level, rather a large gene family, with genome-wide representations. Thus the possible role of sex in CNV among olfactory receptor (OR) gene family was investigated. OR genes (Buck and Axel, 1991; Rouquier et al., 1998) comprise the largest gene family in the human genome, with nearly 900 members (Glusman et al., 2001; Hasin-Brumshtein et al., 2009) and also have been extensively studied, with regard to CNV (Trask et al., 1998; Waszak et al., 2010).

OR gene family is also well-known to have a large number of pseudogenes (about 50%) among its members (Glusman et al., 2001). More recently with newly developed bioinformatics algorithms, many new pseudogenes have been identified in the human genome (Menashe et al., 2006) establishing them as regular constituents of the genomic architecture. Also during the last decade many more studies have indicated specific regulatory functions for pseudogenes (Balakirev and Ayala, 2003; Wen et al., 2011, 2012). Therefore should the results of this study show sex bias among OR pseudogenes, it could be another indication that at least some of them are functional, lending more support to the argument that this large family of genes probably has other functions besides olfaction, like serving as regulatory agents in early embryonic development (Dreyer, 1998).

Using publicly available CNV data for 791 OR loci (Waszak et al., 2010) obtained from 150 phenotypically normal individuals (57 males and 93 females) from the 1000 Genome Project, a total of 3872 CNVs with assigned copy number genotypes (ranging from zero to nine) was extensively analyzed for this population genomics study, which was conducted at three levels: among all 150 individuals, and within and between three diverse populations comprising these 150 individuals. The focal point was to investigate the role of gender in various aspects of CNV among OR gene family, including being more prevalent in pseudogenes or genes, in common or uncommon variants, and in copy number loss or gain. The results portray an unexpectedly complex yet fascinating picture of the effects of gender on the CNV of OR gene family. This complexity entailed some speculations about an indirect role of olfaction system in the development of a bigger brain in humans. Additionally, to examine the clinical relevance of these results, sex bias was assessed in the CNV of children with intellectual disabilities and birth defects, using a public database.

## MATERIAL AND METHODS

### DATA SOURCE

In November 2010, Waszak et al. (2010), in a paper published in PLoS Computational Biology, discussed newly developed software to infer copy number genotype from the next generation sequencing (NGS) data. They benchmarked their prediction algorithm by genotyping 500 chromosome 1 CNV regions in the genomes of 150 people sequenced at low-coverage. The assessed copy number genotypes were highly concordant with their performed qPCR experiments (Pearson correlation coefficient 0.94) and with the published results of two microarray platforms (95–99% concordance). Additionally they used this novel analysis software to assign the copy number genotype for 791 members of OR gene family, located on autosomal chromosomes, using the NGS data of 150 people studied in the 1000 Genome Project.

It should be mentioned that some of the authors of the Waszak et al. (2010) paper have been deeply involved in the OR genomic research for the last two decades, and since 2000 have established and maintained HORDE, the main database for the human OR gene repertoire. The study presented here used the supplementary data, given in Table S4 of Waszak et al. (2010), to analyze the reported CNVs from a new perspective, that is, according to gender. Thus initially two tables of raw data were made, one for the 57 males, and the other for the 93 females of this study, and then each table was analyzed separately.

It is noteworthy to be reminded that they originally detected a total of 4573 CNVs on autosomal chromosomes, but later had to exclude 701 of them as they represented six questionable loci. These loci likely represent extremely rare CNVs that were detected in the reference genome (Waszak et al., 2010). Among 150 individuals of this study, there were 45 CEU (individuals of European ancestry from Utah, USA, including 42 unrelated individuals and 3 members of a parent offspring trio); 52 YRI [unrelated African individuals with ancestry from Nigeria (Yoruba from Ibadan)]; 53 Asians, including 29 unrelated Chinese individuals from Beijing (CHB) and 24 unrelated Japanese from Tokyo (JPT; Waszak et al., 2010).

### STATISTICAL ANALYSIS

The chi-square goodness of fit test was carried out in all but one cases. Expected values were based on the frequencies of the males and females in each tested category. The Fisher's exact test of independence was used in one case, as indicated in the text.

## RESULTS

### SEX BIAS IN CNV AMONG OR PSEUDOGENES AND GENES

Of the 791 autosomal OR loci studied here, 484 (61%) showed no CNV and as expected displayed two copies in all 150 individuals. Among 307 loci that showed CNV in either gender, 177 (58%) were pseudogenes, found on 18 autosomal chromosomes, and 130 (42%) were intact genes, scattered across 13 chromosomes. Although genomic variants, in very large samples, are usually categorized in three groups of very common, less common, and rare (Cirulli and Goldstein, 2010), for this study the 3872 detected CNVs were grouped into two classes, common and uncommon. For the common group, the same criterion as very common was used and the uncommon group included less common and rare variants. This classification was used to more accurately pinpoint the evolutionary history of these CNVs. As generally accepted, the assumption is that the uncommon variants, being less commonly shared, represent more recent events in the genomic evolutionary history.

Common CNV variants were detected in >5% of the individuals, and were found among 57 OR loci, showing CNV in both genders and scattered genome-wide on 12 chromosomes. Uncommon CNVs were those that were detected in 5% or less, that is, in one to eight individuals out of 150. Among the 250 loci showing uncommon variants, there were 63 loci detected in both genders, 94 showing uncommon variants only in the males, and 93 showing uncommon variants only in the females. Obviously with a much larger sample size, many of these loci will show CNVs in both genders.

**Table 1** shows the comparisons of the number of common and uncommon CNV variants between the two genders. Males had significantly more CNVs as a whole and also among both common and uncommon variants. However, the CNV percentage in males showed an increase from 40% (1366/3407) in the common variants to 45% (209/465) in the uncommon variants, whereas the expected percentage was 38%. To better characterize this disparity, OR genes and pseudogenes were studied separately and the results are also given in **Table 1**. As a whole again, the males showed more CNVs among both OR genes and pseudogenes. But when the CNVs detected in genes and pseudogenes were grouped into common and uncommon variants, the results showed that the CNV number in males was very significantly higher than expected only among the uncommon CNV variants of pseudogenes, and not among the common ones (**Table 1**). On the other hand, OR intact genes did not show any significant sex bias in either common or uncommon CNV variants (**Table 1**).

**Table 1:** Gender comparisons of the number of common and uncommon CNV variants among OR genes and pseudogenes.

CNV Category	Gender	Total no. of CNVs	No. of loci	No. of CNVs in pseudogenes	No. of loci	No. of CNVs in genes	No. of loci
Total	In 57 males	1575		1061		514	
	In 93 females	2297		1546		751	
	Total	3872	307	2607	177	1265	130
		*p* = 0.0006		*p* = 0.0045		*p* = 0.0537	
		M > F***		M > F**		M > F*	
Common^a^	In 57 males	1366		928		438	
	In 93 females	2041		1401		640	
	Total	3407	57	2329	34	1078	23
		*p* = 0.0118		*p* = 0.0665		*p* = 0.0752	
		M > F**		n.s		n.s	
Uncommon^b^	In 57 males	209		133		76	
	In 93 females	256		145		111	
	Total	465	250	278	143	187	107
		*p* = 0.0020		*p* = 0.0007		*p* = 0.4567	
		M > F**		M > F***		n.s	

*The chi-square test was carried out in each case. Expected values were based on the total numbers of the males and females in this study, as shown in the second column.*

*M and F refer to male and female, respectively.*

a Common CNV variants were detected in > 5%, that is, in nine to 150 individuals.

b Uncommon CNV variants were detected in about 5%, that is, in one to eight individuals out of 150. Significance level at

* α = 0.05

** α = 0.01

*** α = 0.001

### SEX BIAS WITHIN AND BETWEEN THREE DIVERSE POPULATIONS

#### Absence of sex bias among the East Asian group

It is well-known that diverse populations, with regard to ethnicity, display various degrees of similarities and differences in CNV (Jakobsson et al., 2008; Waszak et al., 2010). Thus to further characterize the significantly higher CNV number seen among the uncommon variants of pseudogenes in the males, as shown in **Table 1**, the 150 individuals of this study were divided into three ethnic groups, based on their ancestries of East Asians (CHB + JPT), Africans (YRI), and Europeans (CEU), as described in Section “Materials and Methods.” And each group was studied separately, and the results are given in **Tables 2** and **3**. With regard to the total number of CNVs, significant sex bias was observed among CEU and YRI groups, in which males showed higher numbers. However, conspicuously no sex bias was detected among the East Asians (**Table 2**). Additionally, only CEU showed significant sex bias for both categories of OR intact genes and pseudogenes (**Table 2**). The same trend in sex bias was also seen among the uncommon CNV variants of OR pseudogenes (**Table 3**), again indicating the absence of sex bias among the East Asians, as well as, the common CNV variants of OR pseudogenes in all three ethnic groups.

**Table 2:** Gender comparisons of the CNV numbers among OR genes and pseudogenes in three diverse populations.

Population designation^a^	Gender	Total no. of CNVs	No. of CNVs in pseudogenes	No. of CNVs in genes
CEU	In 18 males	534	369	165
	In 27 females	662	463	199
	Total	1196	832	364
		*p* = 0.0010	*p* = 0.0104	*p* = 0.0379
		M > F***	M > F**	M > F*
YRI	In 17 males	458	304	154
	In 35 females	827	552	275
	Total	1285	856	429
		*p* = 0.0241	*p* = 0.0782	*p* = 0.1567
		M > F*	n.s	n.s
CHB + JPT	In 22 males	583	388	195
	In 31 females	808	531	277
	Total	1391	919	472
		*p* = 0.7606	*p* = 0.6625	*p* = 0.9308
		n.s	n.s	n.s

*The chi-square test was carried out in each case. Expected values were based on the numbers of the males and females of each ethnic group, as shown in the second column.*

*M and F refer to male and female, respectively.*

a CHB + JPT, Chinese from Beijing plus Japanese from Tokyo; YRI, Yoruba from Ibadan, Nigeria, Africa; CEU, European ancestry from Utah, USA. Significance level at

* α = 0.05

** α = 0.01

*** α = 0.001

**Table 3:** Gender comparisons of the number of uncommon and common CNV variants of OR pseudogenes in three diverse populations.

Population designation^a^	Gender	No. of CNVs in uncommon^b^ pseudogenes	No. of CNVs in common^c^ pseudogenes
CEU	In 18 males	46	323
	In 27 females	37	426
	Total	83	749
		*p* = 0.0041	*p* = 0.0809
		M > F**	n.s
YRI	In 17 males	42	262
	In 35 females	52	500
	Total	94	762
		*p* = 0.0133	*p* = 0.3218
		M > F**	n.s
CHB + JPT	In 22 males	45	343
	In 31 females	56	475
	Total	101	818
		*p* = 0.5346	*p* = 0.8066
		n.s	n.s

*The chi-square test was carried out in each case. Expected values were based on the numbers of the males and females of each ethnic group, as shown in the second column.*

*M and F refer to male and female, respectively.*

a *CHB + JPT, Chinese from Beijing plus Japanese from Tokyo; YRI, Yoruba from Ibadan, Nigeria, Africa; CEU, European ancestry from Utah, USA*.

b *Uncommon CNV variants were detected in about 5%, that is, in one to eight individuals out of 150*.

c *Common CNV variants were detected in >5%, that is, in nine to 150 individuals*.

** *Significance level at α = 0.01*.

#### One-copy loss among OR pseudogenes

To further study the effects of both gender and ethnicity on OR pseudogenes, the total number of CNVs in OR pseudogenes, that is, 2607, was divided into copy number loss and gain as shown in **Table 4A**. The comparisons of CNV number among the three ethnic groups showed a significant population difference only for copy number loss and not gain. On the other hand, OR intact genes did not yield any significant difference in either copy number loss or gain among the three populations (data not shown).

**Table 4:** Gender comparisons of the CNV numbers among OR pseudogenes for one and two-copy number loss between three diverse populations.

		Population designation^a^			Total	No. of loci	*p*-Value	Significance
		CHB + JPT	YRI	CEU				
A.	All	*n* = 53	*n* = 52	*n* = 45	*n* = 150			
	Grand total	919	856	832	2607	177	0.0572	n.s.
	Total gain	396	435	352	1183	86^b^	0.2616	n.s.
	Total loss	523	421	480	1424	104^b^	0.0001	***
	Two-copy loss	260	78	245	583	14	0	***
	One-copy loss	263	343	235	841	104	0.0008	***
B.	Females	*n* = 31	*n* = 35	*n* = 27	*n* = 93			
	Total loss	299	279	266	844	60	0.0223	*
	Two-copy loss	147	57	150	354	12	0	***
	One-copy loss	152	222	116	490	60	0.0013	***
C.	Males	*n* = 22	*n* = 17	*n* = 18	*n* = 57			
	Total loss	224	142	214	580	79	0.0047	**
	Two-copy loss	113	21	95	229	13	0	***
	One-copy loss	111	121	119	351	79	0.0228	*

*The chi-square test was carried out between the three ethnic groups. Expected values were based on the numbers (*n*) of the males and females as given for each group.*

a *CHB + JPT, Chinese from Beijing plus Japanese from Tokyo; YRI, Yoruba from Ibadan, Nigeria, Africa; CEU, European ancestry from Utah, USA*.

b *Total number of OR pseudogenes were 177, but total gain and total loss were 86 and 104, respectively, as there were 13 pseudogenes showing both copy number loss and gain (86 + 104 = 190 - 13 = 177)*. Significance level at

* α = 0.05

** α = 0.01

*** α = 0.001

Since there are two components to copy number loss, losing either one copy or both copies, the total copy number loss was also divided into two parts based on this criterion. As the results in **Table 4A** show, losing either one copy or both copies was significantly different among the three ethnic groups. Next to investigate whether there was also a sex bias in copy number loss between these groups, the number of CNV loss (separately for one copy and two copies) in each group was divided according to gender, and as shown in **Tables 4B,C**, no sex bias was detected.

Interestingly although the loss of one copy did not show any sex bias, among the three populations, for OR pseudogenes (**Table 4**), when pairwise comparison was carried out, significant sex bias was detected between the two populations of 52 YRI and 45 CEU. Interestingly, the results showed no difference among the males of these two groups (**Table 5C**), while their females showed considerable difference (**Table 5B**), as the CEU females had fewer one-copy loss than the YRI females.

**Table 5:** Gender comparisons of the CNV numbers among OR pseudogenes for one-copy loss between two populations.

		Population designation^a^			No. of loci	*p*-Value	Significance
		YRI	CEU	Total			
A.	All	*n* = 52	*n* = 45	*n* = 97			
		343	235	578	88^b^	0.0057	**
B.	Females	*n* = 35	*n* = 27	*n* = 62			
		222	116	338	46	0.0006	***
C.	Males	*n* = 17	*n* = 18	*n* = 35			
		121	119	240	70	0.5672	n.s

*The chi-square test was carried out between the twoethnic groups. Expected values were based on the numbers (*n*) of the males and females as given for each group. Data is from **Table 4***.

a YRI, Yoruba from Ibadan, Nigeria, Africa; CEU, European ancestry from Utah, USA.

b Total number of OR pseudogenes showing one-copy loss among these two populations was 88, but only 46 of them were showing CNV in the females, while 70 in the males. Significance level at

** α = 0.01

*** α = 0.001

The next question was whether there was any sex bias in the number of copy number loss within any of these three populations. The results indicated a significant sex bias in CEU population with regard to one-copy number loss, in which the females had significantly less CNV than the males did, as shown in **Table 6A**. It was an indication that among CEU, the females retained their OR pseudogenes more often than the males did, and as was described in the previous paragraph, the CEU females also showed higher retention of their pseudogenes compared to the females of YRI.

**Table 6:** Within-population sex bias in CNV numbers in each of the three diverse populations.

	Population designation^a^	CNV type	Genomic location	Male	Female	Total	No. of loci	*p*-Value	Significance
				*n* = 18	*n* = 27	*n* = 45			
A.	CEU	One-copy loss	Genome-wide	119	116	235	59	0.0009	M > F***
				*n* = 17	*n* = 35	*n* = 52			
B.	YRI	One-copy loss	OR cluster at chr 11q11^b^	4	53	57	6	0.00004	F > M***
				*n* = 22	*n* = 31	*n* = 53			
C.	CHB + JPT	Gain of three to seven copies	OR clusters at chr 14q11.2 and chr 15q11.2	30	80	110	21	0.0024	F > M**

*The chi-square test was carried out in each case. Expected values were based on the numbers (*n*) of the males and females as given for each ethnic group.*

*M and F refer to male and female, respectively.*

a CHB + JPT, Chinese from Beijing plus Japanese from Tokyo; YRI, Yoruba from Ibadan, Nigeria, Africa; CEU, European ancestry from Utah, USA.

b This cluster was the largest cluster of OR loci, which showed loss of one copy in this study. Significance level at

** α = 0.01

*** α = 0.001

#### Among OR pseudogenes of 7E subfamily

Among 299 human OR subfamilies, 7E is very distinct. First with 86 members, it is the largest subfamily. Secondly all but one of its members are pseudogenes, scattered across most chromosomes (Newman and Trask, 2003; Olender et al., 2008), and third it has undergone extensive human-specific expansion (Feldmesser et al., 2006; Waszak et al., 2010). Also it has been reported to have the highest transcription level among all OR subfamilies (Feldmesser et al., 2006). Chromosomal rearrangements of some segmental duplications containing the 7E pseudogenes have also been implicated in mental retardation and dysmorphic phenotypes (Giglio et al., 2002).

The only gene in this subfamily, *OR7E24*, is located on chr 19q13.2 and did not show any CNV in this study, while among its 40 pseudogenes that showed CNV, a total of 868 CNVs were detected, which was 22% of the total CNVs (868/3872). This high volume was 2× more than expected as the 7E subfamily members make up about 10% of the total number of OR loci. The main reason for this increase was because seven of these pseudogenes had been commonly deleted in the study group, 455 CNVs showing complete deletion and 250 CNVs showing hemizygous deletion (loss of one copy).

As a group, no significant sex bias was detected among the 7E subfamily members (359 CNVs in 57 males vs. 509 in 93 females). However, as this subfamily of pseudogenes exhibited excessive involvement in genomic copy number loss, it was intriguing to know whether gender played any role in this process. Since this study was concerned with more recent OR evolutionary events, and the complete deletion of any gene most likely represents an older evolutionary process, it was decided to concentrate on the 7E loci involved in only the loss of one copy. Thus, 24 pseudogenes were placed under this category, with 116 CNVs.

Then the 116 CNVs were divided based on both gender and ancestry. Comparison among the males of the three populations did not show any significant difference in the number of one-copy loss (**Table 7C**), while there was a significant difference among the females (**Table 7B**). Furthermore, it was noticed that among the 24 pseudogenes, three of them had contributed excessively to the total number of CNVs. After careful inspection, it became evident that 33 out of 34 CNVs detected among the females, for these three loci, came from 35 YRI females. Indeed they were almost the only females who were losing one copy of these three loci (*OR7E97P* on chr 3, *OR7E136P* and *OR7E59P* both tightly linked on chr 7), while 57 out of 58 females with CEU and Asian ancestries all retained their two copies of these three loci. At the same time, loss of one copy of these three loci was seen among the males of all three populations.

**Table 7:** Between-population sex bias in the CNV numbers among 24 pseudogenes of OR7E subfamily that showed strictly one-copy loss.

		Population designation^a^			Total	No. of loci	*p*-Value	Significance
		CHB + JPT	YRI	CEU				
A.	All	*n* = 53	*n* = 52	*n* = 45	*n* = 150			
		24	66	26	116	24	2.50E - 06	***
B.	Females	*n* = 31	*n* = 35	*n* = 27	*n* = 93			
		8	42	9	59	24	6.70E - 07	***
C.	Males	*n* = 22	*n* = 17	*n* = 18	*n* = 57			
		16	24	17	57	24	0.1016	n.s

*The chi-square test was carried out between the three ethnic groups. Expected values were based on the numbers (*n*) of the males and females as given for each group.*

a CHB + JPT, Chinese from Beijing plus Japanese from Tokyo; YRI, Yoruba from Ibadan, Nigeria, Africa; CEU, European ancestry from Utah, USA.

*** Significance level at α = 0.001

#### At chr 11q11

About 50% of all OR loci are located on chr 11, which is also one of the most heavily imprinted chromosomes in the human genome (Taylor et al., 2006). In this study the largest cluster of OR loci showing loss of one copy was located at chr 11q11 @55 Mb, comprising four OR genes and two pseudogenes, and spanning 81 kb. A total of 317 CNVs was detected at this cluster, 276 for loss of one copy and 41 for complete deletion of both copies. To find out whether there was any sex bias at this cluster, the 276 CNVs were divided based on both gender and ancestry for within and between population comparisons. As shown in **Table 8**, there were significant differences among the three populations, both in the males and females, indicating population difference but no sex bias. However, there was a very significant sex bias within the African YRI group, in which only four CNVs were detected in the males (all coming from the same person), while 53 CNVs were found among the females, as shown in **Table 6B**. It is worth mentioning that a recent genome-wide large study has associated the deletion of a region exclusively covering three out of six of these loci, *OR4P4*, *OR4S2*, and *OR4C6* with early-onset (extreme) obesity (Jarick et al., 2011).

**Table 8:** Between-population differences in the CNV numbers for one-copy loss at a OR cluster at chr 11q11^a^.

		Population designation^b^			Total	No. of loci	*p*-Value	Significance
		CHB + JPT	YRI	CEU				
A.	All	*n* = 53	*n* = 52	*n* = 45	*n* = 150			
		131	57	88	276	6	1.10E - 06	***
B.	Females	*n* = 31	*n* = 35	*n* = 27	*n* = 93			
		88	53	46	187	6	0.0003	***
C.	Males	*n* = 22	*n* = 17	*n* = 18	*n* = 57			
		43	4	42	89	6	7.60E - 07	***

*The chi-square test was carried out between the three ethnic groups. Expected values were based on the numbers (*n*) of the males and females as given for each group.*

a This cluster was the largest cluster of OR loci, which showed loss of one copy in this study, see also **Table 6B** for within-population sex bias among YRI.

b CHB + JPT, Chinese from Beijing plus Japanese from Tokyo; YRI, Yoruba from Ibadan, Nigeria, Africa; CEU, European ancestry from Utah, USA.

*** Significance level at α = 0.001.

### SEX BIAS IN CNV BETWEEN SEGMENTAL DUPLICATIONS AT chr 14q11.2 AND chr 15q11.2

The two most CNV-enriched OR clusters in the human genome were located at chr 14q11.2 and chr 15q11.2. Interestingly almost all CNVs were copy number gain. A total of 1560 copy number gain was detected among the 25 OR loci that showed gain in these two clusters, 21 loci with common CNV variants and 4 with uncommon. These clusters were particularly enriched with an extra gene copy (copy number 3) totaling 807 CNVs, 350 in 57 males and 457 in 93 females, and as shown in **Table 9**, the males had significantly more copy number 3 than the females did.

**Table 9:** Sex bias in one-copy gain in two clusters of OR loci at chr 14q11.2 and chr 15q11.2.

Genomic location	CNV type	Male *n* = 57	Female *n* = 93	Total *n* = 150	No. of loci	*p*-Value	Significance
Two clusters of OR loci at chr 14q11.2 and chr 15q11.2	One-copy gain	350	457	807	25	0.0017	M > F**

*The chi-square test was carried out and expected values were based on the total numbers (*n*) of the males and females of this study, as given in this table.*

*M and F refer to male and female, respectively.*

** Significance level at α = 0.01.

On average for every OR locus, about five CNVs were detected in this study (3872 CNVs/791 OR loci). On chr 15q11.12, there was a cluster of eight OR loci, six pseudogenes and two genes, with an average of 87 CNVs per locus (693/8), by far the most CNV-enriched OR cluster in the human genome. Instead of the expected two copies per locus, on average each locus in this cluster had three copies in each individual (3781 total copies/eight loci = 473/150 individuals = 3), regardless of the gender. Notice “total copies” here means total gene copies, and not total CNV number of this region.

Interestingly 99% of the CNVs were involved in copy number gain, from one to seven copies (copy numbers 3–9), and only 1% in copy number loss. This cluster, which spanned about 0.1 Mb, was so polymorphic for CNV that only 3% of 150 individuals did not show any CNV in this cluster. It had very complex copy number structure with many individuals exhibiting a different copy number for each of the eight loci. Interestingly although the males in this study generally showed more CNVs than the females (about 7% more than expected, although not significantly), 31% of the females vs. 26% of the males showed CNV for every locus in this cluster.

It has been suggested that 15q11.2 is a large segmental duplication of the same region at chromosome 14, as they both contain the same OR gene families, OR11 and OR4 (mostly OR4). However, the OR gene cluster at chr 14q11.2, which spanned about 3.4 Mb, was much larger in size and contained 35 loci. But only the first 13 loci, seven pseudogenes and six genes, spanning about 0.5 Mb, showed extensive CNV. After OR cluster at chr 15q11.2, the one at chr 14q11.2 was the most polymorphic CNV cluster, with an average of 66 CNVs for each of 13 loci. The OR loci at these two clusters had very unique CNV anatomy, as they routinely contained copy numbers above four and up to nine. No other OR locus in the human genome showed more than four copies. But the cluster at chr 15q11.2 was even more distinct, as it showed twice as many loci with five to nine copies as the one at chr 14q11.2 (180 vs. 91), even though it had only eight loci showing CNV, compared to 13 at chr 14q11.2.

However, the real variance between these two clusters was the sex bias, which they showed in their CNV profiles. As shown in **Table 10A**, for the 13-loci cluster at chr 14q11.2, with a total of 867 CNVs, the results showed that the males had significantly more CNVs than the females. At the same time, 693 CNVs at chr 15q11.2 cluster of eight OR loci showed no significant sex bias (see **Table 10B**).

**Table 10:** Gender comparisons of the total number of CNVs (loss and gain) between two OR clusters at chr 14q11.2 and chr 15q11.2.

	Genomic location	Male *n* = 57	Female *n* = 93	Total *n* = 150	No. of loci	*p*-Value	Significance
A.	OR cluster at chr 14q11.2^a^	383	484	867	13	0.0002	M > F***
B.	OR cluster at chr 15q11.2^a^	267	426	693	8	0.7745	n.s

*The chi-square test was carried out in each case. Expected values were based on the total numbers (*n*) of the males and females of this study, as given in this table.*

*M and F refer to male and female, respectively.*

a OR clusters at chr 14q11.2 and chr 15q11.2 are the two most CNV-enriched OR clusters in the human genome.

*** Significance level at α = 0.001.

One reason for the failure of the males to show higher number of CNV at chr 15q11.2 cluster was due to the uniqueness of copy number 5 profile (having five copies at any given locus) at this cluster, in which it was present significantly more in the females than males (observed values 68 vs. 22; expected values 56 vs. 34; *p* = 0.0081, using chi-square test). On the other hand the incidence of five copies among the seven OR pseudogenes at chr 14q11.2 was exactly as expected, that is, 29 CNVs in the females vs. 18 in the males. It should be noted that the CNV dynamics at these two clusters were very complex and unpredictable, except for the loci at chr 15q11.2 that all consistently showed higher frequency of copy number 5 in the females.

There was yet another significant result with regard to having higher copy numbers, ranging from five to nine, among females. The East Asian group, which as a whole did not show any sex bias in this study, showed a significant sex bias, in opposite direction, for higher copy numbers, in which the females showed higher CNVs in 5–9 copy range (**Table 6C**). When compared with the corresponding numbers in the other two groups, it became apparent that the CNVs was more or less as expected in the Asian males, while the Asian females showed excessive CNVs for 5–9 copies (31 and 33 CNVs among 17 males of YRI and 18 males of CEU, respectively; 53 and 44 CNVs among 35 females of YRI and 27 females of CEU, respectively).

## DISCUSSION

Copy number variation is a major form of genomic structural variation, in which any given locus or a large segment of DNA has a different copy number than the expected number. They could occur either in germ cells, or later during early embryonic development, through both recombination-based and replication-based mechanisms (Hastings et al., 2009) and they tend to be overrepresented among genes with olfactory, immunity, and secretory functions (Nguyen et al., 2006). With regard to OR repertoire, its genes and pseudogenes comprise the largest gene family in the human genome, with nearly 900 members (Glusman et al., 2001; Hasin-Brumshtein et al., 2009), making up about 1% of the genome.

Over a decade of intense international research has implicated CNV in the etiology and development of various genetic disorders and many types of cancer (McCarroll and Altshuler, 2007; Sebat et al., 2007; Cook and Scherer, 2008; Ionita-Laza et al., 2009; Conrad et al., 2010; Chen et al., 2011a,^b^). With regard to the OR gene family, at population level, Young et al. (2008)reported extensive CNV among OR loci, which played a considerable role in the genomic diversity among some 50 individuals tested in their study. And the same year, Hasin et al. (2008) by examining CNV in 25 individuals, created a high resolution map of CNV affecting OR genes, and used this map for inferring the evolution of OR gene repertoire. Then in 2010, Waszak et al. (2010) provided exact copy number genotypes for about 800 OR genes in 150 individuals, using a novel computational approach on NGS data. This approach makes CNV data exceedingly accessible for population studies, and they reported some differences in CNV among various human populations.

The study presented here is the first study revealing sex bias in CNV of olfactory gene family. In 2011, there was also a report of finding a sex-specific association of CNV at chr 6p21.3 with nasopharyngeal carcinoma susceptibility (Tse et al., 2011). Sex bias in single-nucleotide polymorphism (SNP) has also been reported recently for tissue factor (TF) and TF pathway inhibitor (TFPI) genes among patients with coronary heart disease and type-2 diabetes (Opstad et al., 2010) and the risk of breast cancer in men (Orr et al., 2011). As indicated, all these three reports were involved in specific patient cohorts. However, Mittelstrass et al. (2011) studying 3300 individuals, from a general population living in Southern Germany, reported extensive sexual dimorphism in 102 out of 131 serum metabolite concentrations, and also described finding sex bias in SNPs of an enzyme central to amino acid metabolism. Very recently Dimas et al. (2012) reported human regulatory variation in females and males and demonstrated that 12–15% of autosomal expression quantitative trait loci (eQTLs) function in a sex-biased manner.

In this study, analysis of 3872 CNVs of 307 OR loci among 150 individuals revealed that males had significantly more CNVs than females (**Table 1**). Since CNVs are suggested to occur through recombination, and recombination is known to have different rates among males and females, one can assume that sex-specific recombination could be responsible for the observed sex bias. But most recombination events are meiotic, occurring during germ cells production. And germ cells are passed from one generation to the next. However, the required differential rate of copy number loss and gain of OR loci, among the two genders, would be best explained through a mechanism that would not entail the passage of these newly made CNVs from one generation to the next. One such mechanism would be differential retrotransposition that could occur during embryonic development or even after birth. Although OR pseudogenes are not processed pseudogenes, that is, were not originated through retrotransposition of their intact OR genes, there is no reason to assume that their subsequent deletion or duplication could not occur through this process. One way to test this proposition would be the examination of CNVs in parents and their offspring to see whether they are inherited or made *de novo*, or both.

Additionally, the males showed higher number of CNVs in all four categories that were tested in this study for OR loci: OR genes, OR pseudogenes, plus common and uncommon variants of OR loci. However, when the combined effects of any two categories of functionality and commonality were studied, the sex bias in CNV was found to be significant only among the uncommon CNV variants of OR pseudogenes (**Table 1**). This novel finding could imply the gender's more recent evolutionary role in shaping the human genomic architecture of autosomal chromosomes.

However, an important point needs to be deliberated here, as currently the line between the OR genes and pseudogenes is getting considerably blurry, due to the recent availability of DNA sequences of OR loci in an increasing number of individuals. In 2003, 26 OR genes were reported as being segregating pseudogenes (Menashe et al., 2003) containing SNPs which rendered one allele functional and the other non-functional (pseudogene). Later three more segregating pseudogenes were added to this list (Young et al., 2008). And more recently this number has increased substantially. In a study of 651 individuals from the 1000 Genome Project, investigating 413 intact OR loci, 244 of them were identified as being segregating pseudogenes, with both intact and pseudogene forms found in this population (Olender et al., 2012). Thus in the near future with the availability of more data to define the exact frequencies of various segregating pseudogenes in different populations, it will be a more appropriate time to reevaluate the current finding with regard to sex bias in the uncommon CNV variants of OR pseudogenes.

Furthermore, in this study several cases of sex bias in OR copy number, involving both copy number loss and gain, were presented at three population levels: among 150 phenotypically normal individuals (93 females and 57 males) from the 1000 Genome Project (**Tables 1**, **9**, and **10A**), between the three diverse populations that these individuals came from (**Tables 2**, **3**, **5**, and **7**), and within each of the three populations (**Tables 2**, **3**, and **6**). The comparisons were carried out by exploring the OR gene family as a whole, which is scattered across the entire genome, as well as, four sub-genomic categories: OR7E subfamily, the largest OR subfamily, made up almost exclusively of 85 pseudogenes; an OR cluster at chr 11q11, harboring the highest concentration of OR one-copy loss in this study; and two OR clusters at chr 14q11.2 and chr 15q11.2, comprising the two most OR CNV-enriched regions in the human genome. These two clusters were also distinct by showing almost no OR copy number loss and instead harboring the gain of higher copy numbers ranging from five to nine (gain of three to seven copies), detected nowhere else in the genome among OR loci. Nevertheless, the search for finding sex bias was not exhaustive, and many other OR families/subfamilies and genomic regions remain to be further explored, preferentially with a larger sample size.

### ETHNICITY AND DIFFERENTIAL RETENTION OF OR PSEUDOGENES AMONG THE GENDERS

In this study several cases were presented showing sex bias among OR pseudogenes, which under the assumption of non-functionality of pseudogenes is expected to occur equally in both genders. A prominent case involved the females with European ancestry (CEU), consistently showing less one-copy loss of OR pseudogenes, when compared with either the males of CEU or the females of YRI (**Tables 5B** and **6A**). Taken together it seemed that these females were retaining their pseudogenes more than others did. With regard to the loss of both copies, more males and females of YRI retained their OR pseudogenes, when compared to the members of the other two groups (**Tables 4B,C**), which was detected in eight out of 14 pseudogenes showing loss of both copies. In this case the individuals from the other two groups were losing these eight loci very commonly (in >20% of them), while the YRI group was not losing them as often, or losing them only sporadically.

Interestingly as the results in **Tables 2** and **3** show, sex bias had quite different magnification among the three ethnic groups. It was noticeably prominent in CEU group, less so in YRI (but still statistically significant), and generally absent in CHB + JPT. There was only one case in this study that showed sex bias among the East Asian group, in which the females rather than the males showed higher copy number gain, exclusively seen at two OR clusters on chr 14 and chr 15 (**Table 6C**). The sex bias trend among these three groups, as seen here, follows suspiciously the general observation that sexual dimorphism, mostly with regard to secondary sex characteristics, are most visible among the Caucasians and least so among the people of East and Southeast Asia. Whether this similarity represents a causal or incidental association is by itself an interesting subject matter worthy of further research.

Evolutionary speaking, differential retention of some OR pseudogenes among the genders could be selectively neutral or under rather recent selective pressure. Also it might not be due to the outside selective pressure from olfactory stimuli, as pseudogenes have no direct olfactory functions. Rather it might be due to internal embryonic competition between the maternal and paternal genomes for the control of monoallelic transcription of some OR pseudogenes. Thus they might have some parent-of-origin regulatory functions. Also as shown in **Tables 1** and **3**, since the strongest sex bias was observed among the uncommon CNV variants of OR pseudogenes, it might point to the relatively recent regulatory role of gender in this process.

In recent years several studies have implicated pseudogenes as having active regulatory functions by turning into non-coding small RNAs and causing interference (Sasidharan and Gerstein, 2008). In a review, Balakirev and Ayala (2003) summarized the studies carried out in *Drosophila* and other organisms that indicated functional roles for many pseudogenes. Another study in mouse has implicated a functional role for an expressed pseudogene in regulating the mRNA stability of its homologous coding gene (Hirotsune et al., 2003). And recently, a cluster of small interfering RNAs (siRNAs) derived from the pseudogenes of African *Trypanosoma brucei* have been reported to suppress gene expression through RNA interference (Wen et al., 2011).

### COMPLETE DELETION OF OR PSEUDOGENES AND ITS POTENTIAL APPLICATION IN HUMAN DNA IDENTITY

Most cases of sex bias in CNV detected in this study were involved in loss of just one copy of OR pseudogenes (**Tables 5**, **6A,B**, and **7**). In general 59% of all OR pseudogenes (104/177) showed one-copy loss, accounting for about 32% of all CNVs in OR pseudogenes (841/2607). Among these loci, 17% were commonly losing one of their two copies (18/104), while the rest were sporadically losing one copy. It should be emphasized that not all these 104 loci showed sex bias in CNV.

With regard to the loss of both copies of OR pseudogenes, it accounted for 22% of their CNVs (583/2607, **Table 4A**). However, only 14 OR pseudogenes were involved, comprising about 8% of all OR pseudogenes that showed CNV (14/177) and all of them were common deletions. Interestingly as shown in **Table 4A**, the number of these deletions was very significantly different between the three populations, with no sex bias being detected, as both genders also showed significant variations between the populations.

The significant population differences in the number of deletions of OR pseudogenes could point to the presence of population-specific deletions among the three populations that potentially could have applications in paternity and forensic complex cases, in which extra genomic data would be needed to exclude certain suspects. After careful inspection of the 14 loci involved in complete deletion, five pseudogenes from OR7E sub family looked very promising. Since in this study there were considerably more females than males (93 vs. 57) only the female's results are considered here. (1) There were two loci, *OR7E86P* and *OR7E84P*, on chr 4 that had high rates of complete deletion among CEU and CHB + JPT females, while complete deletion was not detected at all among 35 YRI females. (2) There were three loci, *OR7E140P*, *OR7E148P*, and *OR7E149P* that were totally absent in 27 CEU females, while detected in the females of the other two groups. Clearly a much larger sample size is needed to verify these two conclusions.

### PWS/AS BI-DIRECTIONALLY IMPRINTED REGION AND THE MOST POLYMORPHIC CNV-ENRICHED CLUSTER OF OR LOCI IN THE HUMAN GENOME

The most CNV-enriched cluster of OR loci in this study was located on chr 15q11.2, consisting of eight OR loci, six pseudogenes and two genes, as detailed extensively in the Section “Results.” Looking at the CNV data of this region seemed like gazing at the epicenter of genomic seismic activity with regard to recombination and/or retrotransposition events. This cluster is part of a large segmental duplication in chr 15q11.2, but the CNV dynamics in this cluster were very complex, especially when all eight loci showed CNVs, ranging mostly from three to nine copies. In a typical segmental duplication one expects to see all loci showing more or less the same copy number, unless it was ancient enough to have been broken down by recombination events numerous times. Alternatively it might not be an ancient genomic region, just a target of many recent retrotranspositions, or most likely a composite of both events.

The most intriguing part of this cluster was in its gender profile with regard to CNV. As shown in **Table 10B**, among 693 CNVs in this cluster, no sex bias was detected. Whereas in a comparable OR cluster, a cluster of 13 OR loci at chr 14q11.2 with 867 CNVs (the second most CNV-enriched OR cluster, per locus, in the human genome), the results showed that the males had significantly more CNVs than the females (**Table 10A**). One main reason for the males not showing higher CNVs at chr 15q11.2 was due to the fact that having five copies of any given locus was significantly more prevalent among the females than the males in this region, thus offsetting the males' general higher CNVs (see Results). Having five copies or more was exclusively observed for the OR loci located in these two clusters. So the fact that the females showed more five copies at chr 15q11.2, and not for chr 14q11.2, might imply some biological significance.

The OR cluster at chr 15q11.2 is located 1.4 Mb proximal to Prader–Willi syndrome/Angelman syndrome (PWS/AS) bi-directionally imprinted region, involved in two well-known but rare human genetic disorders, PWS and AS, associated with developmental delay and mental retardation (Mann and Bartolomei, 1999; Horsthemke and Wagstaff, 2008). This region is one of the most, if not the most polymorphic, dynamic piece of real estate in the human genome (Chen et al., 2011a,^b^) sitting next to one of the most valuable pieces of genomic real estate, a large cluster of tandem repeats of ribosomal RNA genes (rDNA), one of five such clusters that constitute nucleolus organizer regions (NORs), on the short arm of five acrocentric chromosomes. The NORs are the regional factories that assemble ribosomes, the genomic power houses that later are transported to cytoplasm to make proteins for the cells. It should be noted here that the human chromosome 2 is most likely a reconstruction of two such acrocentric chromosomes still present in orangutan. It is well-known that the q11.2 regions of both chr 14 and chr 15 are the grave yards of many types of pseudogenes, and OR pseudogenes are not the only ones populating these regions (GeneCards V3). The amassing of pseudogenes at the q11.2 region is also noticeable for chr 21, and chr 22, which are also acrocentric chromosomes, containing NOR.

Prader–Willi syndrome in most cases is the result of a 6 Mb deletion of the paternal copy of chr 15q11–q13 region. In the majority of other cases, maternal uniparental disomy (UPD) of chr 15, or microdeletions in a regulatory region known as the imprinting center have been identified in PWS (Jiang et al., 2008). The deletion of the maternal copy of the same region in majority of cases causes AS, as well as paternal UPD. PWS and AS critical regions, sitting next to each other contain several coding and non-coding genes (Ohta et al., 1999) that have been the target of intense research, but nevertheless the identity of the exact gene or non-coding RNA is debated. But most consider a defect in UBE3A, and the deletion of 29 copies of small nucleolar RNA (snoRNA) as the causal agents in AS and PWS, respectively. In a normal individual, this region is bi-directionally imprinted; one section of this region is expressed normally in the chromosome coming from a specific parent, while the other section is imprinted, and vice versa for the chromosome coming from the other parent.

Chr 15q11.2 is a very unstable region of the human genome, as evident by the deletions observed in the patients with PWS and AS. This region on the one hand harbors a bi-directional imprinted region, and on the other hand a unique polymorphic cluster of OR loci, with regard to its exceedingly large numbers of CNVs and also the gender profile of its CNVs. Are these two incidences independent of each other? Could the OR cluster have a unique CNV profile because it is next to an imprinted region? Or could this region be imprinted because it is next to a unique OR cluster? Further research should shed some light on any potential causal relationship between the PWS/AS imprinted region and the CNV gender profile of the OR cluster at chr 15q11.2.

Initially it could be argued that olfaction would not have any role in mental retardation. But the epigenetic causes of mental retardation, as a developmental delay disorder, are particularly complex. Olfaction in most mammals is not only related to the sense of smell but also primitive cognition. As such, during human early embryonic development, from an evolutionary perspective, the human genome, as a mammalian genome, arguably has to switch from an evolutionarily more primitive cognitive stage, heavily based on olfaction and pheromones, to a newer cognitive stage, primarily based on a larger, thinking brain. Although a large number of OR genes and vomeronasal receptor genes (putative pheromone receptors) have turned into pseudogenes in primates, still the embryonic olfaction assembly must be under a specific regulatory system that would need to be precisely adjusted at a specific embryonic stage in primates to allow a new pathway starts its journey of making a bigger brain. At this critical stage, any genetic or epigenetic perturbation could cause delay in this time-sensitive process, resulting in general developmental delays, including mental retardation.

Therefore, mental retardation, as the word “retardation” implies, is basically a developmental delay disorder, which might be caused by sex-specific dis-regulation of the timing of suppression of olfaction system and its OR pseudogenes. Is there any evidence to remotely support this notion? One of the most well-known clusters of genes that are temporally regulated during embryonic development sits in a sea of OR loci on the short arm of chr 11. The human beta globin locus, composed of five genes (epsilon, gamma-G, gamma-A, delta, and beta) is temporally regulated by locus control region (LCR), which is located closest to the epsilon gene, the first one to be expressed at the earliest embryonic stage. Among these genes and surrounding these genes, there are over 100 OR loci, containing all class I (fish-like receptor) OR genes and pseudogenes (OR families 51–56). In a very recent study, the same LCR has been shown to control more than 1000 promoters and regulatory elements genome-wide (Stamatoyannopoulos et al., 2012). Thus it is quite possible that the OR loci of this region or those on other chromosomes would be also under the temporal regulation of this LCR.

### CLINICAL APPLICATION OF SEX BIAS IN CNV

As in this study sex bias in CNV was shown only among the OR loci of normal people, it poses two new questions, that is, whether it exists genome-wide among other loci, and whether abnormal sex bias in CNV could be associated with genetic disorders. The answer to these questions are beyond the limits of this study. But since there is already ample evidence for sex bias in clinical presentations of many genetic disorders (Neul and Zoghbi, 2004; Rinehart et al., 2011) in this section the association between sex bias in CNV and genetic disorders will be examined, without inferring whether the sex bias was normal or abnormal. The unraveling of any association could greatly bring into focus the past and current genome-wide association studies and as will be discussed here, it also could advance clinical molecular diagnostics, especially for kids with syndromic, as well as, idiopathic mental retardation.

In 2010 American College of Medical Genetics (ACMG) recommended replacing G-banded karyotyping with chromosomal microarray analysis (CMA) as the new standard practice for genetic evaluation of children with unexplained developmental delay, intellectual disability, autism, or birth defects. In the CMA test, certain chromosomal rearrangements, like microdeletions and microduplications are detected in each patient's DNA sample, in the form of CNV. Each CNV is defined by the exact chromosomal location of its DNA sequence and its corresponding cytogenetic band.

Currently about 10% of these CNVs are assigned as pathogenic, about 7% VOUS (variant of unknown significance) indicating that this variant has not been detected in the phenotypically normal people, but there is no information available about its pathogenicity. The remaining 83% CNVs are of no use to the ordering physicians, as they are assigned benign CNVs. Following the International Standard Cytogenomic Array Consortium (ISCA) protocol (Miller et al., 2010), this large portion of CNVs are assigned benign by analysis software, after searching the Database of Genomic Variants (DGV) for similar CNVs in phenotypically normal individuals, as currently enlisted in 42 publications, and managed by DGV.

One way to drastically improve the usefulness of the current CMA test is to better characterize the potential role of these presumed benign CNVs in the pathogenicity of disorders related to intellectual disability. Although they are found to be common, it does not necessarily mean that they could not be pathogenic in certain circumstances. For example some of them could have some imprinting defects, as the CMA test would not check the genomic imprinting status. Also in specific ethnic groups, in combination with certain genes, they might prove to be pathogenic.

A simple approach to improve the current CMA test would be to check any sex bias among all detected benign CNVs in each tested patient. Currently there are at least two databases that provide patients' CMA test results in addition to their clinical presentations. For clarity an example of a benign CNV will be presented here. *Dusp22* (dual specificity phosphatase 22) is a gene at the most telomeric region of Chromosome 6, at p25.3. This gene was selected here because copy number loss and gain are commonly seen for this gene in many CMA test results, and also for evolutionary consideration, as the cytogenetic band 6p25.3 is much longer in chimps and gorillas, compare to humans and orangutans.

Searching the ISCA public database for *Dusp22*, 96 males and 32 females showed CNVs for this gene (as of February 14, 2012). It should be added that these CNVs were not only for this gene, rather any loss or gain also containing this gene. So deletions and duplications from 1.9 Mb to 33.6 kb were reported for the chromosomal region containing this gene. Out of 128 patients, in 126 cases, the related CNVs were reported as benign, as their CNVs were also detected in the phenotypically normal individuals. In the other two cases, one female had about 2 Mb gain at this region, and thus reported as pathogenic, and one male with about 1.2 Mb loss had an “uncertain” diagnosis.

Although in this example, the number of the males was 3× of the females, since the total number for the male and female patients were not provided in this database, it would not be accurate to conclude that there were significantly more males showing CNV for *Dusp22*. However, the number of loss and gain provided for each gender could easily overcome this shortcoming, as the real issue here is finding sex bias in the number of loss and gain. For the males there were 72 cases of copy number gain, and 24 cases of loss, while for the females there were 10 cases of gain and 22 cases of copy number loss. The Fisher's exact test of independence showed significant gender bias between copy number loss and gain for this region (**Table 11**). In other words, the males compared to the females had significantly more copy number gain than loss in the *Dusp22* region.

**Table 11:** Very significant sex bias between copy-number gain and loss for a genomic region containing *Dusp22* gene at chr 6p25.3, among children with developmental delay disorders and dysmorphic phenotypes.

	Copy number gain	Copy number loss	Total
Males	72	24	96
Females	10	22	32
Total	82	46	128
			*p* = 0.00001

*The Fisher's exact test of independence was carried out for this comparison*.

*The data is from ISCA public database (as of February 14, 2012)*.

As for their clinical presentations, the ISCA database indicated that among the 31 females, two were reported having autism phenotype (both with copy number loss). However, among the 95 males, a proportionally higher number, that is, 12 were reported with autism phenotype,(10 with copy number gain). Further examination of the genomic region showing copy number loss and gain in these kids, pointed to a small region of 80 kb, covering Dusp22 and its immediate vicinity, in all 12 cases.

There are two other concerns that need to be addressed. First whether the other 83 males (95 - 12) of this group, with no autism diagnosis, shared CNV-enrichment in the same 80 kb region or not. And the next concern would be whether the same sex bias in copy number loss and gain would be detected in the phenotypically normal people with the same ethnicity. The second question could be answered by using publicly available databases specifically designed to be used as controls (Shaikh et al., 2009). Thus for many genetic disorders that show sex bias at the level of clinical presentation (including several intellectual disabilities), this simple method holds promise for easily locating a candidate chromosomal region for further comprehensive study, including comparison of the epigenetic state of the candidate region among the patients and their controls.

## Conflict of Interest Statement

The author declares that the research was conducted in the absence of any commercial or financial relationships that could be construed as a potential conflict of interest.
